# A Rare Case of Incarcerated Inguinal Hernia Containing Fat and a Penile Reservoir

**DOI:** 10.7759/cureus.34315

**Published:** 2023-01-28

**Authors:** Luiza Miziara Brochi, Raul Mederos, Mohammed Al Bashir

**Affiliations:** 1 General Surgery, Universidade de Uberaba, Uberaba, BRA; 2 Surgery, Hialeah Hospital, Hialeah, USA; 3 General Surgery, University of Khartoum, Khartoum, SDN

**Keywords:** hernia repair, reservoir displacement, penile implant complication, penile prothesis, incarcerated inguinal hernia

## Abstract

The inflatable penile prosthesis (IPP) is a three-piece device indicated to treat erectile dysfunction. Although it is considered a safe procedure, it can result in complications, such as reservoir herniation. Literature is scarce regarding reservoir incarcerated herniation as a complication of IPP and its management. Surgery is required to reduce symptomatic hernias and properly secure the reservoir to avoid recurrence. An untreated incarcerated hernia may lead to strangulation and necrosis of abdominal organs, as well as implant malfunction. We present a rare case of a left-sided incarcerated inguinal hernia containing fat and a penile reservoir of a previous penile prosthesis implant in a 79-year-old man, as well as the technique used to correct it.

## Introduction

The inflatable penile prosthesis (IPP) is the most common surgical treatment for erectile dysfunction (ED) and is usually indicated in pharmacologically refractory cases. The device is composed of dual intra-corporal inflatable cylinders, a scrotal pump, and a fluid reservoir [1,2]. It is often performed after radical prostatectomy because ED is a possible complication of this procedure [3]. IPP placement is considered safe, and it has the highest satisfaction rates of all ED treatment options, exceeding 95% in postoperative questionnaires [2]. Although complications of this treatment are rare, they are a possibility and can negatively impact patients’ quality of life [4]. Reservoir herniation is a very unusual condition, and literature is scarce in detailed reports on the presentation and management of these cases, especially regarding incarcerated inguinal hernias containing the implant’s reservoir.

We present a rare case of a left-sided incarcerated inguinal hernia containing omental fat and a fluid reservoir of a previous IPP in a 79-year-old man, as well as the detailed technique used to correct it.

## Case presentation

A 79-year-old male presented to Urgent Care with a complaint of moderate intermittent pain in the left lower quadrant of the abdomen for three days. He shared that this had been a recurrent problem, with no other associated symptoms. His surgical history was significant for a penile prosthesis implant, which was performed at another service six years prior to this visit. A physical examination of the abdomen showed a non-reducible left inguinal hernia. The patient’s vital signs showed a heart rate of 66 beats per minute, respiratory rate of 16 breaths per minute, axillary temperature of 36.4°C, and blood pressure of 131/88 mmHg.

**Figure 1:**
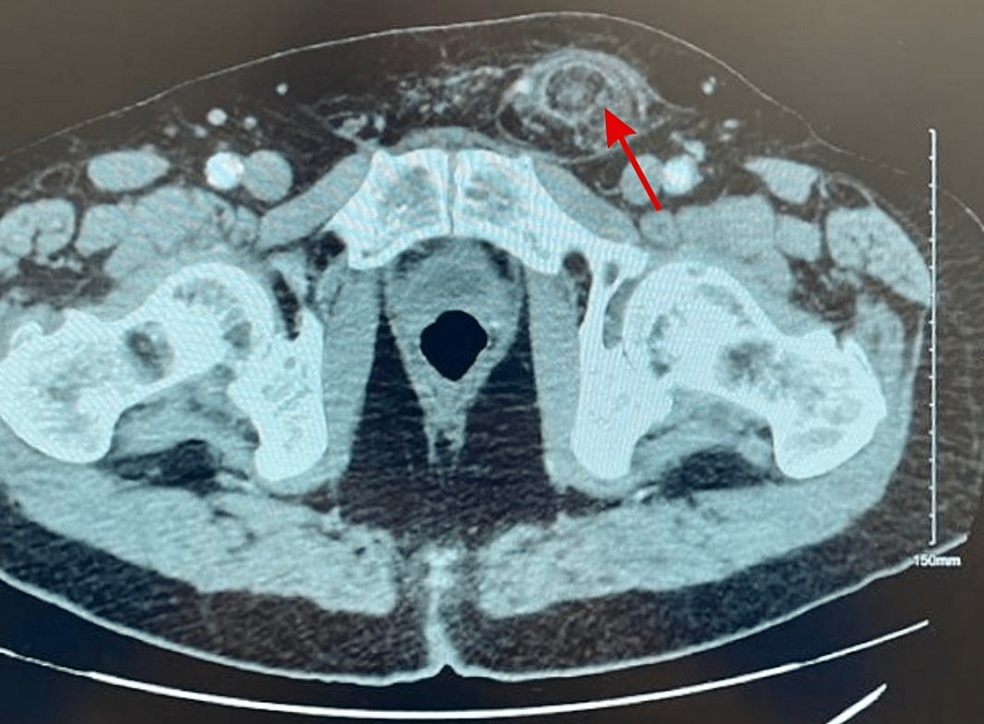
CT scan of the pelvis and lower abdomen. Axial plane of the CT scan. The red arrow indicates the left inguinal hernia.

**Figure 2:**
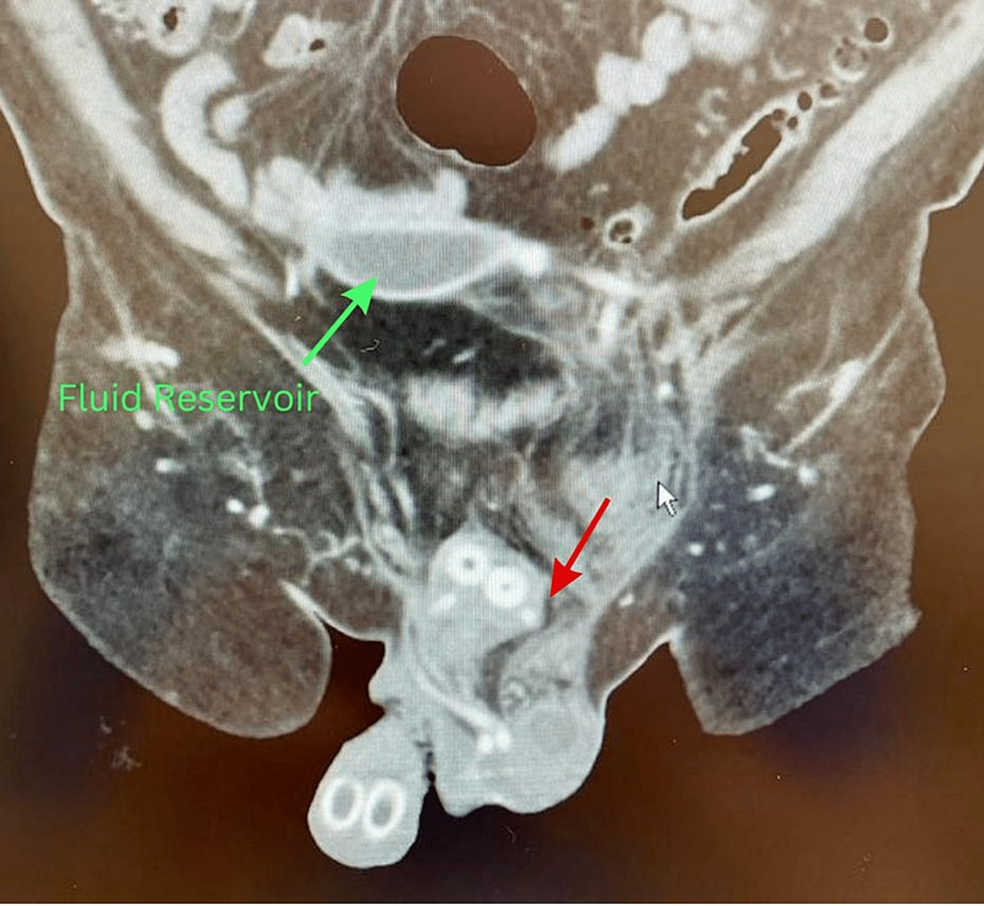
CT scan of the pelvis and lower abdomen. Sagittal plane of the CT scan. The red arrow indicates the left inguinal hernia, and the green arrow indicates the fluid reservoir in the abdominal cavity.

A decision was made to approach this case with imaging studies. A pelvic computed tomography (CT) scan with contrast showed a fat-containing 8 cm left inguinal hernia (Figures 1, 2). Fat stranding and fluids in the hernia sac were seen, which suggested acute inflammation possibly due to vascular compromise. A left inguinal ultrasound was also performed, which showed a 7 × 4.1 × 6.6 cm heterogeneous structure in the left inguinal region with trace surrounding fluid. The patient was transferred to the Emergency Room with a diagnosis of incarcerated left inguinal hernia (ICD K40.30).

**Figure 3:**
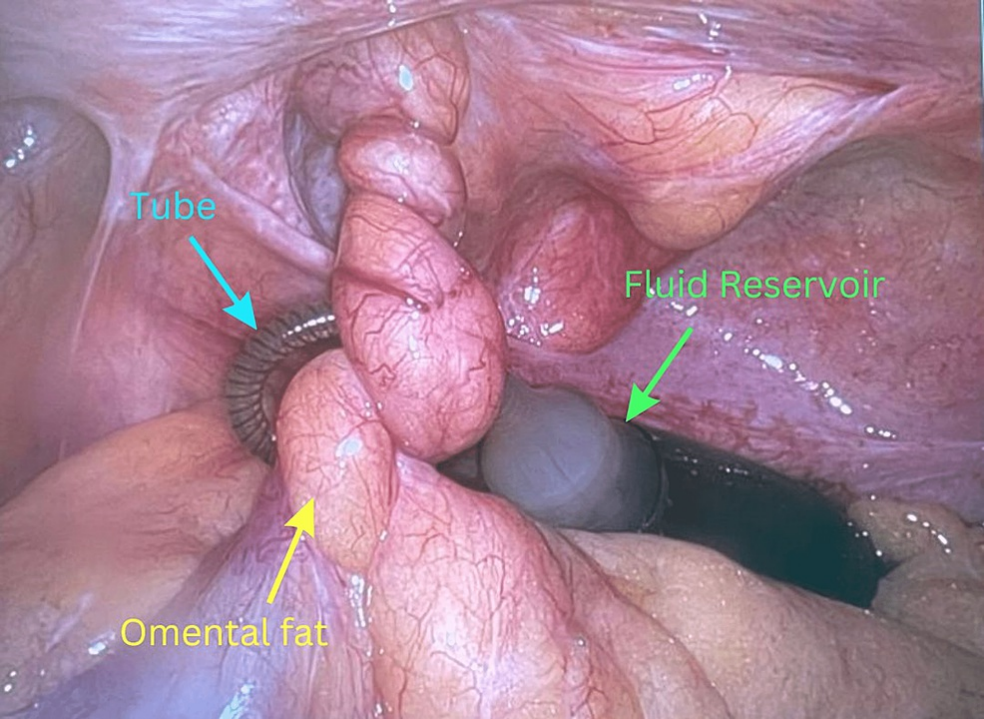
Robotic repair of the incarcerated hernia. Intraoperative image of the robotic transabdominal preperitoneal hernia repair. Omental fat (yellow arrow) is trapped around the fluid reservoir (green arrow) and the tube (blue arrow) inside the abdominal cavity.

**Figure 4:**
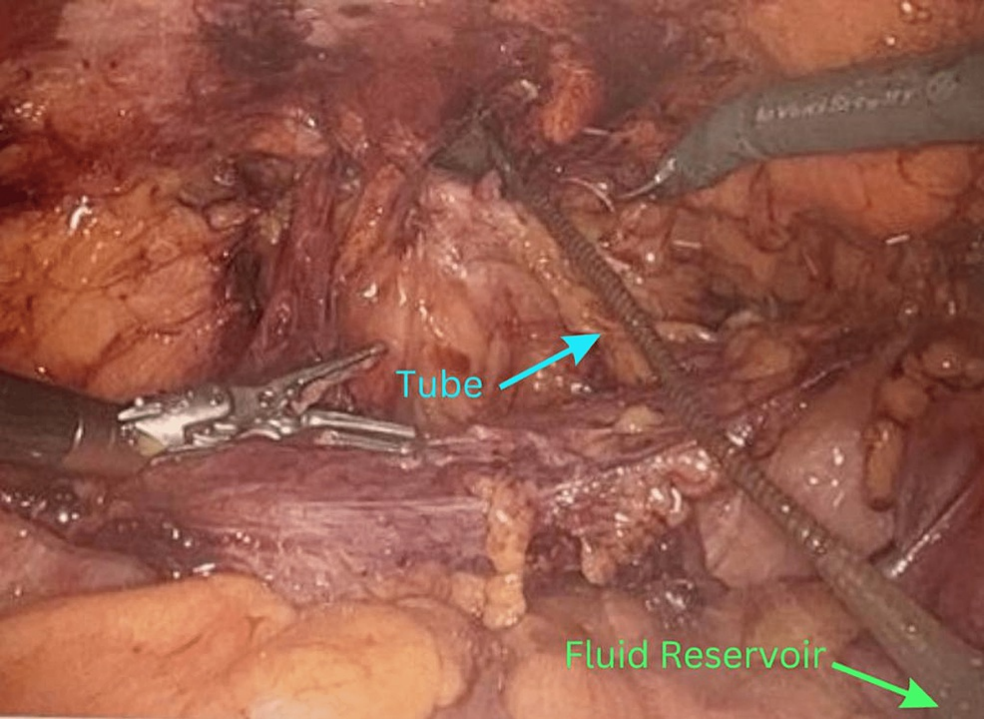
Robotic repair of the incarcerated hernia. Intraoperative image of the robotic transabdominal preperitoneal hernia Repair. Untangled tube (blue arrow) and reservoir (green arrow) inside the abdominal cavity.

**Figure 5:**
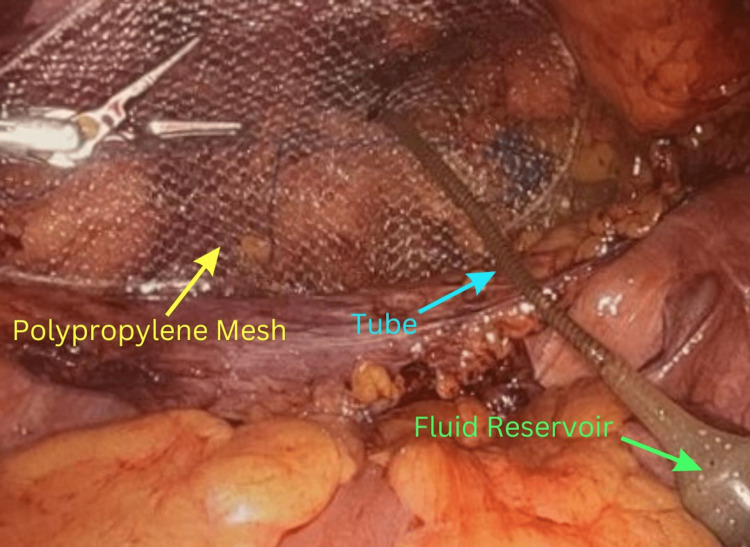
Robotic repair of the incarcerated hernia. Intraoperative image of the robotic transabdominal preperitoneal hernia repair. Mesh (yellow arrow) placed underneath the pelvic muscles, tube (blue arrow), and reservoir (green arrow) inside the abdominal cavity.

The patient was taken to the operating room for a robotic transabdominal preperitoneal repair of the incarcerated hernia. A direct incarcerated inguinal hernia was encountered on the left side. The reservoir from his previous surgery was located inside the abdominal cavity, and there was a piece of omental fat trapped within the hernia sac that was entangled with the tubing that connected the reservoir to the pump of the penile implant (Figure 3). It was possible to separate the structures and reduce the incarcerated hernia completely inside the abdominal cavity (Figure 4). The incarcerated fat appeared necrotic and was removed. A polypropylene mesh was applied to decrease the risk of recurrence (Figure 5). The reservoir was placed between the mesh and the peritoneum, which was then reflected up and sutured together covering both the mesh and the reservoir completely. The patient tolerated the procedure well without any complications. He had an uneventful recovery and was sent home on postoperative day number one. He was seen in the clinic the following week and was recovering well.

## Discussion

Complete herniation of the IPP reservoir is an extremely unusual event, and literature is scarce in presenting detailed case reports of such complications, especially regarding incarcerated hernias, such as the one reported in this study.

In the literature, the first record of inguinal hernia associated with penile implant malfunction is from 1998 in a series of two cases. One of the patients presented with a disruption of the tubing that connected the reservoir to the pump, and the other had the reservoir migrated to the scrotum. Both were associated with right inguinal hernias, and both had previous pelvic surgery; however, in contrast to our case, neither of these hernias was incarcerated [5].

Sadeghi-Nejad et al. reviewed a database of 1,206 IPPs and found an overall herniation incidence of 0.07% to the inguinal canal or to the scrotum. Unlike in our patient, they were mostly associated with the immediate postoperative period and acute increase in intra-abdominal pressure (such as vomiting or coughing) [6].

A cohort study of 246 IPPs performed by a single urologist reported two cases of inguinal hernia in a single patient with previous pelvic surgery. Different from our case, the hernia sac contained bowels but had no reservoir involvement. In addition, five other cases of inguinal hernias were reported in patients with no history of pelvic surgery. Of those, one hernia sac contained only bowels, and two contained partial involvement of the reservoir; the other two were asymptomatic and the doctor decided to follow up conservatively, therefore, they were not submitted to surgery [7].

There is no consensus in the literature about whether previous pelvic surgery is a major risk factor for developing herniations. Most authors affirm that to be true because invasive procedures may cause adhesions, weakening of the pelvic muscles, and distortion of pelvic anatomy [8]. However, in the study by Madiraju et al., no significant difference was noted when comparing the incidence of negative outcomes in both groups [9]. Nevertheless, many surgeons recommend moving the reservoir from its traditional impalpable position in the space of Retzius to an alternative place, such as between the rectus abdominis musculature and transversalis fascia, or between the transversalis fascia and the peritoneum [10]. In our case, the patient reported having prostate surgery years before, which was confirmed by the CT scan results, which showed no visible prostate.

Many IPP placement techniques have been proven to be successful, and yet there is no standard technique to assess this complication. It is evident that appropriate IPP indication, methodical preoperative assessment, and specific intraoperative and postoperative measures may contribute to decreasing the risk of adverse events [11].

## Conclusions

Even though IPPs are considered safe procedures with a very low rate of non-infectious complications, adverse outcomes should be studied because they are a possibility. This knowledge is fundamental to optimizing surgical outcomes and patient satisfaction.

We recommend assessing the risk factors for reservoir herniation (such as previous pelvic surgery or trauma and chronic increase of intra-abdominal pressure) before indicating any penile implant procedures. Moreover, we recommend a management approach that will also assure that the penile implant will continue to do its function normally without the need for any additional surgery.
